# Sequences, sequence clusters and bacterial species

**DOI:** 10.1098/rstb.2006.1917

**Published:** 2006-10-06

**Authors:** William P Hanage, Christophe Fraser, Brian G Spratt

**Affiliations:** Department of Infectious Disease Epidemiology, Imperial College London, St Mary's Hospital CampusNorfolk Place, London W2 1PG, UK

**Keywords:** multilocus sequence analysis, bacterial populations, species clusters, electronic taxonomy, bacterial systematics

## Abstract

Whatever else they should share, strains of bacteria assigned to the same species should have house-keeping genes that are similar in sequence. Single gene sequences (or rRNA gene sequences) have very few informative sites to resolve the strains of closely related species, and relationships among similar species may be confounded by interspecies recombination. A more promising approach (multilocus sequence analysis, MLSA) is to concatenate the sequences of multiple house-keeping loci and to observe the patterns of clustering among large populations of strains of closely related named bacterial species. Recent studies have shown that large populations can be resolved into non-overlapping sequence clusters that agree well with species assigned by the standard microbiological methods. The use of clustering patterns to inform the division of closely related populations into species has many advantages for poorly studied bacteria (or to re-evaluate well-studied species), as it provides a way of recognizing natural discontinuities in the distribution of similar genotypes. Clustering patterns can be used by expert groups as the basis of a pragmatic approach to assigning species, taking into account whatever additional data are available (e.g. similarities in ecology, phenotype and gene content). The development of large MLSA Internet databases provides the ability to assign new strains to previously defined species clusters and an electronic taxonomy. The advantages and problems in using sequence clusters as the basis of species assignments are discussed.

## 1. Introduction

Bacterial species exist … bacterial diversity is organized into discrete phenotypic and genetic clusters, which are separated by large phenotypic and genetic gaps, and these clusters are recognized as species. Fred Cohan (2002)

To many microbiologists, bacterial species are real entities that can be recognized as clusters of genotypes which are clearly resolved from similar clusters (Palys *et al*. 1997). In fact, there are almost no data that address this assertion, which in essence is a statement of belief. A more agnostic view is to ask whether populations of similar bacteria do invariably (or usually) form discrete well-resolved genotypic clusters that merit the status of species and to consider which methods should be employed to address this issue (Hanage *et al*. 2005*a*). The question is of some importance to microbial taxonomists. If species exist as well-resolved genotypic clusters, they could be defined more naturally, and microbial taxonomists could move forward from their present practice, which is largely based on defining species by rules and cut-off values that take no account of any natural discontinuities in the clustering of related genotypes.

In this article, we examine whether sequences can be used to resolve clusters of related genotypes among large populations of similar bacteria. The focus is not on theoretical considerations, but on the practical issue of whether gene sequences can inform the process by which closely related bacteria are assigned to species, and the prospects for an electronic taxonomy in which bacterial strains can be assigned to species on the Internet (Gevers *et al*. 2005). Reviews of species concepts and definitions as they apply to bacteria can be found in other articles in this volume (Cohan 2006; Staley 2006) and elsewhere (Vandamme *et al*. 1996; Ward 1998; Rossello-Mora & Amann 2001; Cohan 2002; Stackebrandt *et al*. 2002; Gevers *et al*. 2005).

The idea that there are real entities within the bacterial world which we can assign as species is so pervasive (and useful) that it is difficult to avoid using the term. In this paper, we use ‘species’ both in the abstract sense of coherent groups of organisms into which we wish to divide the bacterial world, and to describe real groups of organisms assigned (often inconsistently) as named species by microbiologists. We also refer throughout to bacterial species, but the issues and approaches apply equally to the archaea.

## 2. From rules and cut-off values to sequence clusters

Species delineation is presently based on genetic relatedness using DNA–DNA hybridization as a proxy measure; strains that show approximately 70% or greater DNA–DNA relatedness are considered to belong to the same species and those that have less than this value are different species (Wayne *et al*. 1987). Perhaps surprisingly, given the remarkable and unexpected variation in gene content among strains of the same species (Welch *et al*. 2002), this criterion has worked well and has been supported by recent studies that relate the percentage of DNA–DNA hybridization to measures of average nucleotide identity among all the shared genes using pairs of strains whose genomes have been sequenced (Konstantinidis & Tiedje 2005).

Variation in gene content and the extent of sequence diversity among strains assigned by DNA–DNA hybridization to the same species have led some to consider that current species are too broad, compared to those in higher organisms (Staley 1997), whereas in other cases, it has been suggested that the 70% DNA–DNA relatedness criterion may need to be relaxed for defining some species (Vandamme *et al*. 1996). This, together with difficulties in applying the DNA–DNA hybridization to large numbers of bacteria, the inability to apply it to bacteria that so far are unculturable and discomfort with relying on guidelines, rules and cut-off values, has led to a search for a more natural and more convenient approach to species definition (Gevers *et al*. 2005).

Comparisons of DNA sequences provide a possible way of distinguishing species. The most common and the most generally applicable approach is the use of 16S rRNA sequences, as these can be obtained from both culturable and unculturable bacteria, and sequencing provides precise digital data that can be held in a single database, which can be interrogated via the Internet (Cole *et al*. 2003). Strains exhibiting more than 70% DNA–DNA hybridization (or which have more than 94% average nucleotide identity over all shared genes) have been shown to be extremely similar in their 16S rRNA gene sequences (Konstantinidis & Tiedje 2005). However, the converse is not necessarily true; strains that have almost identical 16S rRNA sequences may not be closely related generally, whether this is judged by DNA–DNA hybridization (Rossello-Mora & Amann 2001) or the average nucleotide identity between all the shared genes (Konstantinidis & Tiedje 2005). Two strains with 16S rRNA sequences that are less than 97% identical are therefore assigned with high confidence to different species, but DNA–DNA hybridization is still required to establish whether strains that have 97% or more 16S rRNA similarity should or should not be placed in the same species (Vandamme *et al*. 1996).

The 16S rRNA sequences have been widely used in environmental microbiology and have been invaluable for uncovering the vast diversity of microbial life (DeLong & Pace 2001), and for assigning unculturable organisms as new species (Hugenholtz *et al*. 1998; Garrity *et al*. 2002), and more problematically, have been used (either implicitly or explicitly) to define species (e.g. studies of species richness or diversity in the oceans; Schloss & Handelsman 2004; Thompson *et al*. 2005*a*). Species definition using rRNA sequences is problematic as this slowly evolving molecule lacks the required level of resolution to distinguish similar species or to address the question of whether species exist and can be clearly resolved (Fox *et al*. 1992). Relationships inferred from 16S rRNA genes may also be distorted by recombination among similar species, which further complicates their use in species definition (Smith *et al*. 1999; Boucher *et al*. 2004).

**Figure 1:**
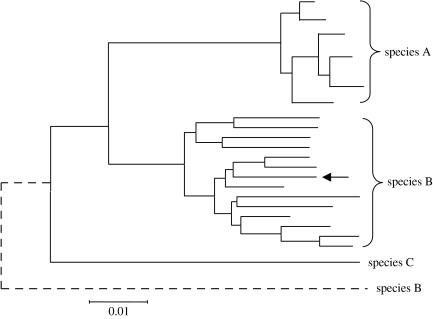
Interspecies recombination and its effect on species assignments based on a single gene sequence. The relatedness among isolates of three species is inferred from a tree constructed using the sequences of a single house-keeping gene. Isolates of species A are well resolved from those of species B, and from the strain of the more distantly related species C used an outgroup. Consider a homologous recombinational event that occurs in a strain of species B (arrow), replacing the single locus used to assign the species with the corresponding sequence from a strain of a relatively divergent species. Now, the strain will not be recognized as a strain of species B and will be incorrectly assigned (dotted line) as more distantly related to species B than the outgroup.

The sequences of a number of protein coding genes have also been used to assign bacteria to species. These have the advantage over 16S rRNA of evolving more rapidly and thus provide an increased ability to resolve species within a genus. However, the use of a single gene has major drawbacks as there may be too few informative nucleotide sites to resolve very similar species and homologous recombination (HR) among similar species may distort the true relationships between species (figure 1). HR is believed to be relatively common among many bacteria (Feil & Spratt 2001) and probably also among many archaea (Papke *et al*. 2004; Whitaker *et al*. 2005). It typically results in the replacement of a small fragment (a few kilobases) of the chromosome of a recipient bacterium with the corresponding region from a different strain of the species or from a closely related species. Laboratory studies indicate that the efficiency of recombination falls off in a log-linear fashion with increasing sequence divergence (Majewski & Cohan 1999; Majewski *et al*. 2000), but there is ample evidence that replacements occur in nature between species that differ at 5–25% in nucleotide sequence (Dowson *et al*. 1989; Spratt *et al*. 1989; Zhou *et al*. 1997; Kalia *et al*. 2001).

The deficiencies of using a single gene to resolve similar species can be overcome by the use of multiple gene sequences, as this approach provides more informative nucleotide sites and also buffers against the distorting effects of recombination at one of the loci (Hanage *et al*. 2005*a*). A homologous interspecies recombinational replacement involving one locus, introduced from species B into a strain of species A, should not prevent the strain being correctly assigned to species A, as the combined sequences of the other loci should still result in it clustering with other strains of species A. Analysis of the relatedness of strains of similar species using multiple gene sequences hence provides the most appropriate way of identifying genotypic clusters and of evaluating the ability of sequences to resolve species (Gevers *et al*. 2005).

## 3. Resolving species using multiple house-keeping genes

Several papers have recently addressed whether the sequences of multiple genes can be employed to distinguish similar species, to inform the division of a genus into species, or to ask whether bacterial species exist (Godoy *et al*. 2003; Priest *et al*. 2004; Baldwin *et al*. 2005; Hanage *et al*. 2005*a*,*b*; Thompson *et al*. 2005*b*). The house-keeping genes are used for this purpose as they evolve relatively slowly (though more rapidly than 16S rRNA genes) and most of the variation that accumulates in these genes is considered to be selectively neutral. Furthermore, house-keeping genes encode products that are likely to be essential to the bacteria and consequently are expected to be present in all strains of a genus.

The basic approach is to concatenate the sequences from the multiple house-keeping loci, use the concatenated sequences from a set of strains to construct a dendrogram and observe the patterns of clustering of genotypes. The number of genes that should be used and the size of the fragments of each gene that should be sequenced have not been systematically explored. More loci presumably increase the ability to resolve genotypic clusters, but exploring patterns of clustering within a genus, or whether resolved clusters exist, requires the analysis of large numbers of strains (discussed later). As a compromise between the need for both resolution and practicality, approximately seven loci are probably adequate, but this may be increased if necessary. Although this approach has not yet been reported, it may be an advantage when setting up a multilocus sequence analysis (MLSA) scheme to analyse clustering patterns on an initial subset of strains using more than seven loci, so that if the evolutionary history of any locus appears to be sufficiently anomalous to disrupt the relationships inferred using all the other loci, it can be identified (e.g. Falush *et al*. 2006) and omitted from the final set of chosen loci.

The use of seven loci has become the norm for characterizing strains within a bacterial species by multilocus sequence typing (MLST; Maiden *et al*. 1998). In MLST, each different sequence is assigned a different allele number and each strain is defined unambiguously by seven integers, which correspond to the allele numbers at the seven loci (the allelic profile). The relatedness among the strains of a species is displayed as a dendrogram using the matrix of pairwise differences in their allelic profiles. In some cases, strains of several closely related species have been characterized by MLST, using the same set of seven genes, and have allowed an analysis of the relationships between species (Godoy *et al*. 2003; Baldwin *et al*. 2005; Hanage *et al*. 2005*a*,*b*). Strains of different species will almost invariably have different alleles at all seven loci, and therefore, the concatenated sequences of the seven loci are used to explore the relationships among the strains of similar species. This extension of the MLST approach has been termed MLSA (Gevers *et al*. 2005).

The MLSA approach has increasingly been used to establish the phylogenetic position of new species (e.g. Christensen *et al*. 2004; Holmes *et al*. 2004) and the relationships between species in closely related genera (e.g. Wertz *et al*. 2003). A few studies have used the approach to look at the relationships among the closely related species within a single genus. Thus, MLSA has been used to resolve the species and candidate species in the *Burkholderia cepacia* species complex (Baldwin *et al*. 2005) and to resolve *Vibrio* species (Thompson *et al*. 2005*b*). These studies have shown resolution of the named species, but have only used small numbers of each species. Bacteria exist as populations and addressing the relationships among similar species requires the analysis of large populations, to see if species clusters can be resolved, to circumscribe the limits of these species clusters and to explore the extent of separation between them. A more rigorous test is therefore to examine whether MLSA can resolve large numbers of strains of very similar species into non-overlapping clusters.

Three recent examples of the use of MLSA to resolve species clusters are discussed here. It should be stressed that in these examples, trees are used solely to establish the patterns of clustering and not to infer the phylogenetic relationships between clusters or of the strains within individual clusters. Trees have been constructed using MrBayes v. 3.1 (Ronquist & Huelsenbeck 2003) with priors for the best-fitting model of nucleotide substitution determined using MrModeltest v. 2.2 (Nylander *et al*. 2004). In the case of the *Neisseria*, a smaller dataset composed of the first 100 *Neisseria meningitidis* and *Neisseria lactamica* strains and all 67 *Neisseria gonorrhoeae* strains was used to obtain this as a result of excessive computational time for calculations using the whole dataset.

## 4. Resolving *Burkholderia pseudomallei*, *Burkholderia mallei* and *Burkholderia thailandensis*

**Figure 2:**
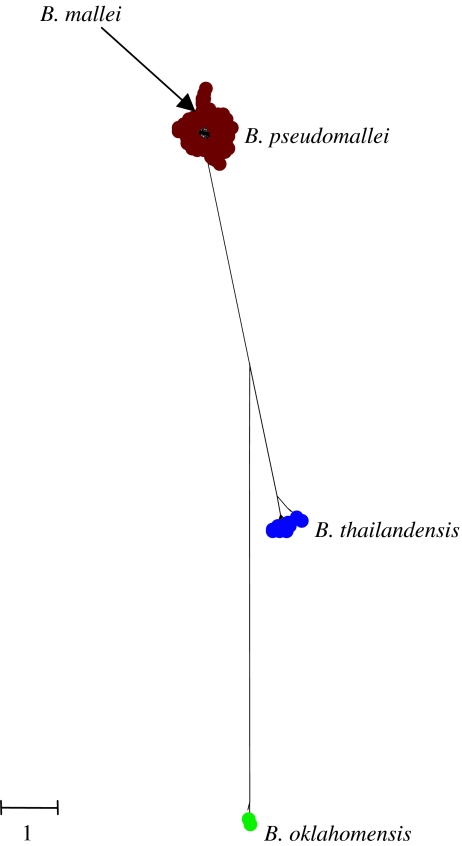
Resolving populations of *B. pseudomallei*, *B. mallei* and *B. thailandensis*. All of the isolates in the *B. pseudomallei* MLST database (which includes isolates of closely related species) were extracted and the sequences at the seven MLST loci were concatenated for each different multilocus genotype (strain) and a tree was constructed using MrBayes v. 3.1. The dataset included 400 different strains (STs) of *B. pseudomallei*, 17 of *B. thailandensis*, and two each of *B. mallei* and *B. oklahomensis*. The scale shows genetic distance, corrected for the best-fitting substitution model determined using MrModeltest and MrBayes. All nucleotide sites were used in the analysis. A general time reversible model was implemented with rate matrix *r*(A↔C) 0.012: *r*(A↔G) 0.419: *r*(A↔T) 0.020: *r*(C↔G) 0.024: *r*(C↔T) 0.509: *r*(G↔T) 0.016; nucleotide frequencies A 0.18: C 0.35: G 0.32: T 0.15 and gamma parameter *α*=0.11. Pinvar=0.82. All trees and model parameters are based on 10 000 samples from the posterior probability at stationarity.

Godoy *et al*. (2003) characterized strains of *Burkholderia pseudomallei*, *Burkholderia mallei* and *Burkholderia thailandensis* by MLST, and examined the resolution of these species using the MLSA approach. The clustering patterns can be re-examined using the current *B. pseudomallei* MLST database (http://bpseudomallei.mlst.net), which now includes 770 isolates of *B. pseudomallei*, 36 of *B. mallei* and 24 of *B. thailandensis*. A tree constructed from the concatenated sequences of the seven MLST loci from one example of each of the 421 different multilocus genotypes (strains) in the current database shows that all *B. pseudomallei* are tightly clustered and are well resolved from a second cluster, which includes all *B. thailandensis* (figure 2). Both of these named species are soil saprophytes and are very closely related, but can be distinguished phenotypically by whether or not they can assimilate arabinose, and clinically by the fact that *B. pseudomallei* can cause serious disease following inoculation or inhalation, whereas *B. thailandensis* is considered to be avirulent.

Although the average sequence divergence between *B. pseudomallei* and *B. thailandensis* is only *ca* 3%, the MLSA approach supports the view that these should be considered as separate species as they are well resolved on the tree (posterior probability of each node is found to be 100%), and there is no sharing of alleles between the species. Isolates of the third species, *B. mallei*, the cause of glanders (primarily a disease of equines, and occasionally of humans), cluster within *B. pseudomallei*. All 36 *B. mallei* in the current *B. pseudomallei* MLST website, recovered from worldwide sources over a period of 40 years, are identical by MLST (excepting one isolate that differs at only a single nucleotide site in one of the seven loci). This ‘species’ can therefore be considered to be a strain (or clone) of *B. pseudomallei* that has been historically given a separate species name by medical microbiologists due to the fact that it causes a distinctive disease (glanders), which differentiates it from *B. pseudomallei*, which is the cause of melioidosis.

The MLSA analysis of Godoy *et al*. (2003) also resolved the taxonomic status of a strain tentatively assigned as *B. pseudomallei* (Yabuuchi *et al*. 1992) that was recovered from a patient involved in a tractor accident in Oklahoma (a non-endemic area for this species). This strain was shown by MLSA to be clearly distinct from both *B. pseudomallei* and *B. thailandensis* (figure 2), and subsequently, a second similar strain has been identified in the United States and these have formally been assigned to a new species, *Burkholderia oklahomensis* (Glass *et al*. 2006).

## 5. Species within species

*Burkholderia mallei* is an interesting example of ‘species within a species’, which highlights some of the problems in bacterial species assignment. By MLSA, it is unambiguously a clone of *B. pseudomallei*; alleles at six of the seven loci of *B. mallei* are also present in *B. pseudomallei* and the seventh differs from a *B. pseudomallei* allele at only a single nucleotide site (Godoy *et al*. 2003). However, it has a genome that is at least one megabase smaller, a different ecology, no longer surviving in soil and depending upon transmission among equines, and therefore almost certainly is on a different evolutionary trajectory (in fact, *B. mallei* is probably on a trajectory to extinction as glanders is now extremely rare and restricted to a few endemic foci in Asia, Africa and the Middle East). The MLSA approach has identified other examples, where historically, bacteria associated with a distinctive human or animal disease have been given species names, but in reality are a clone (or a cluster of closely related clones) with distinctive biology and ecology within a ‘mother species’ (e.g. *Bacillus anthracis* and *Salmonella typhi*; Kidgell *et al*. 2002; Priest *et al*. 2004). These anomalies are probably the inevitable consequence of the recent emergence of distinctive lineages that are destined to emerge as new species from within an existing species.

## 6. Resolving similar recombining species that share the same niche

A particularly challenging test of the existence of species is to examine patterns of clustering among large populations of similar bacteria in which recombination is known to be frequent, and to occur between the species, and where the bacteria colonize the same body site and thus have opportunities to exchange genes. Two recent studies have applied MLSA to ask whether distinct genotypic clusters can be resolved among co-colonizing recombinogenic populations.

The human *Neisseria* species provides the first example (Hanage *et al*. 2005*a*). The taxonomy of the genus *Neisseria* has been the subject of considerable revision over the years and includes two pathogens—*N. meningitidis* and *N. gonorrhoeae*—and a number of human commensal *Neisseria* and several animal species. *Neisseria meningitidis* is commonly carried asymptomatically in the human nasopharynx, but can occasionally gain access to the blood and cerebrospinal fluid to result in septicaemia and meningitis. *Neisseria gonorrhoeae*, which causes gonorrhoea, is closely related to *N. meningitidis*, and is primarily recovered from the human genital tract, although it may also be recovered from the rectum and naso- or oropharynx. House-keeping genes of *N. gonorrhoeae* are much more uniform in sequence than those of *N. meningitidis* and it has been suggested that the former pathogen arose relatively recently as a strain of a human nasopharyngeal *Neisseria* species that acquired the ability to colonize the genital tract and to be transmitted by the sexual route (Vázquez *et al*. 1993).

The other human *Neisseria* species (e.g. *N. lactamica*) are all colonizers of the nasopharynx and are considered to be non-pathogenic commensals, although some have occasionally been associated with disease. These named commensal species therefore may coexist along with *N. meningitidis* within an individual human nasopharynx, providing opportunities for recombination within and between the closely related species. The public *Neisseria* MLST database (http://pubmlst.org/neisseria) contains the sequences of seven house-keeping genes from many thousand strains of *N. meningitidis* and much smaller numbers of *N. lactamica* and *N. gonorrhoeae* strains. We extracted from the public *Neisseria* MLST database the sequences of the seven MLST loci of 500 different strains of *N. meningitidis*, 171 strains of *N. lactamica* and 67 strains of *N. gonorrhoeae*, and used the concatenated sequences of the seven loci to explore the patterns of clustering and to examine the relationships between the observed clusters and the species names assigned by standard microbiological procedures (Hanage *et al*. 2005*a*).

**Figure 3:**
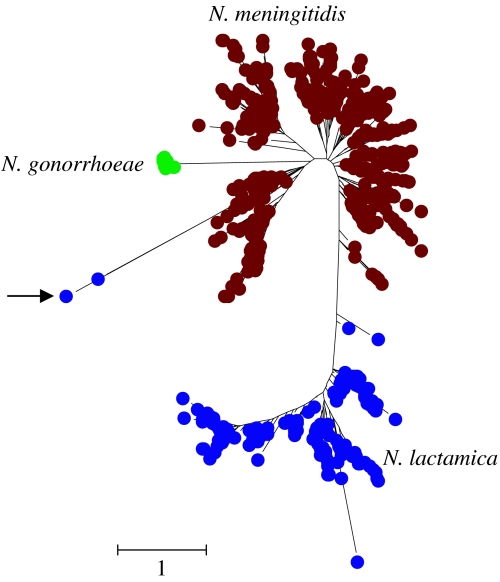
Resolving populations of *N. meningitidis*, *N. meningitidis* and *N. gonorrhoeae*. Bayesian tree constructed using the concatenated sequences (seven loci) of the first 500 different strains (STs) of *N. meningitidis* in the public *Neisseria* MLST database, all different strains of *N. lactamica* (171) and *N. gonorrhoeae* (67). The arrow shows the two strains of *N. lactamica* that cluster anomalously and have probably been incorrectly identified (see text). Only third codon positions were used in the analysis. The scale shows genetic distance, corrected for the best-fitting substitution model determined using MrModeltest and MrBayes. Details as in figure 2 with rate matrix *r*(A↔C) 0.044: *r*(A↔G) 0.541: *r*(A↔T) 0.018: *r*(C↔G) 0.044: *r*(C↔T) 0.299: *r*(G↔T) 0.053; nucleotide frequencies A 0.11: C 0.44: G 0.24: T 0.21 and gamma parameter *α*=0.481. Pinvar=0.30.

Analysis of the sequences of *N. meningitidis* house-keeping genes has shown extensive evidence for recombinational imports from related commensal species (Feil *et al*. 1995; Zhou *et al*. 1997), and the trees of different *Neisseria* house-keeping genes (and 16S rRNA genes) suggest different phylogenetic relationships between these species (Smith *et al*. 1999). As expected, the individual trees derived from the sequences of each MLST locus fail to resolve *N. meningitidis* strains from *N. lactamica* strains (Hanage *et al*. 2005*a*). However, the concatenated sequences of the seven MLST loci completely resolve the *N. lactamica* strains from those of *N. meningitidis* (the group of strains shown as *N. lactamica* were clustered together in 100% of trees drawn from the posterior probability), although a few strains arise from the branch separating these two species (figure 3), and two strains located at the end of a long branch arising from the meningococcal cluster are clear examples of mistaken identity (arrow in figure 3). When examples of other *Neisseria* species are included, these two ‘*lactamica*’ strains cluster with these (Hanage *et al*. 2005*a*) and they have probably been misidentified as *N. lactamica* by the submitting microbiological laboratory. Strains of the ecologically isolated species, *N. gonorrhoeae*, form a tight genotypic cluster on a long branch (100% support) and are closely allied to *N. meningitidis*.

Recombining species that colonize the same body site can therefore be resolved using MLSA, but recombination between similar species can lead to strains ‘creeping’ along the branch that separates the species clusters. If we knew nothing about these strains, it would not be clear where we should put the dividing line between the *N. meningitidis* and *N. lactamica* clusters. Recombining species can therefore appear fuzzy and it may be difficult using MLSA to unambiguously assign a few strains to one species rather than the other. Furthermore, it would not be clear that the *N. gonorrhoeae* cluster should be considered to be distinct from *N. meningitidis*. These results stress the need for MLSA to be used as the basis for pragmatic decisions by expert groups about where to draw distinctions between species, and the need to map onto the observed patterns of clustering whatever additional information is available. In this way, the special nature of the strains within the *N. gonorrhoeae* cluster would be very apparent from their different ecological niche and disease association.

The second challenging example is provided by *Streptococcus pneumoniae* (the pneumococcus) and its closest known relatives (Hanage *et al*. 2005*b*). Pneumococci asymptomatically colonize the human nasopharynx, but occasionally can cause pneumonia, septicaemia or meningitis, and more commonly, acute otitis media and sinusitus. Pneumococci recovered from disease are usually encapsulated (serotypable) and can be identified by their reactivity with typing sera directed against the capsular polysaccharide. These serotypable isolates are usually susceptible to optochin and bile soluble, and isolates with these characteristics are considered unambiguously to be *S. pneumoniae*. Within the nasopharynx, there are a number of organisms that appear to be very similar to pneumococci, but which are non-serotypable, and often may be optochin resistant and/or bile insoluble (Arbique *et al*. 2004). The relationship of these isolates (usually called atypical pneumococci) to authentic pneumococci has been unclear (Whatmore *et al*. 2000).

**Figure 4:**
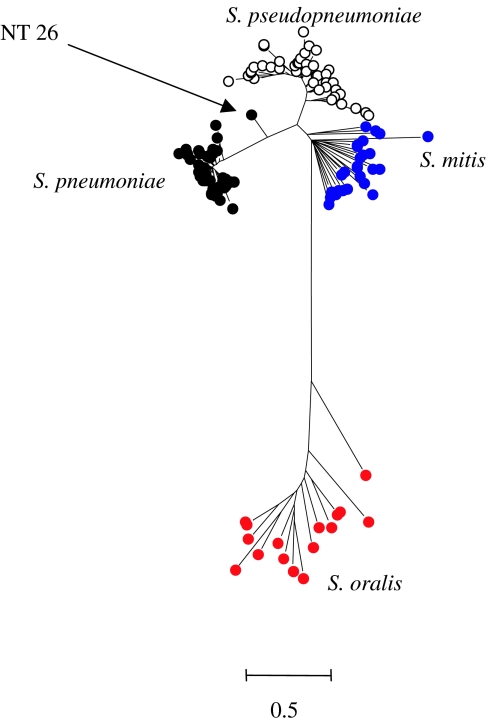
Resolving populations of *S. pneumoniae*, *S. pseudopneumoniae*, *S. mitis* and *S. oralis*. Bayesian tree constructed using the concatenated sequences of six of the MLST loci of the authentic pneumococci and atypical pneumococci (now called *S. pseudopneumoniae*; Arbique *et al*. 2004) studied by Hanage *et al*. (2005*b*), and strains assigned as *S. mitis* and *S. oralis*. NT26 is a non-serotypable presumptive pneumococcus that arises from the branch leading to the *S. pneumoniae* cluster. The scale shows genetic distance, corrected for the best-fitting substitution model determined using MrModeltest and MrBayes. All nucleotide sites were used in the analysis. Details as in figure 2 with rate matrix *r*(A↔C) 0.016: *r*(A↔G) 0.027: *r*(A↔T) 0.010: *r*(C↔G) 0.001: *r*(C↔T) 0.939: *r*(G↔T) 0.007; nucleotide frequencies A 0.31: C 0.18: G 0.24: T 0.27 and gamma parameter with a covarion model allowing rates to change across the tree *s*(off→on)=0.33 and *s* (on→off)=1.33.

A tree constructed from the concatenated sequences (six loci) from 39 different serotypable strains of *S. pneumoniae*, representing the diversity among more than 2000 different strains in the pneumococcal MLST database (http://spneumoniae.mlst.net), and 121 atypical pneumococci showed a clear resolution into two clusters (Hanage *et al*. 2005*b*). One cluster includes all the serotypable pneumococci and a subset of the atypical pneumococci, and another cluster includes the remaining atypical pneumococci. The former class of atypical pneumococci are almost certainly pneumococci that for various reasons are not expressing a capsular polysaccharide, whereas the latter group appear to be a very closely related but distinct population (Hanage *et al*. 2005*b*). As in the *Neisseria* example, there was a good separation of the clusters (100% posterior probability), but some ‘fuzziness’ as one non-serotypable strain arose from the branch separating the two clusters (figure 3). Recently, Arbique *et al*. (2004) have also concluded (using DNA–DNA hybridization) that a subset of atypical pneumococci should be assigned to a different species, *Streptococcus pseudopneumoniae*. MLSA of the two reference strains of this new species obtained from these authors shows that *S. pseudopneumoniae* corresponds to the cluster in figure 4 that is similar to, but distinct from, authentic pneumococci.

**Figure 5:**
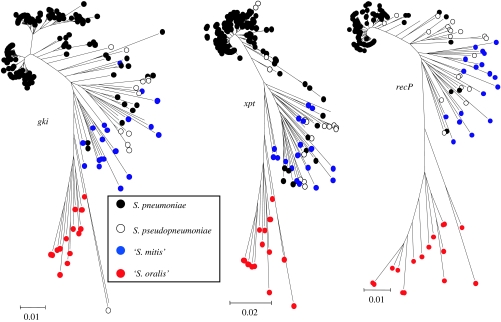
Failure of single loci to resolve *S. pneumoniae* and related species. The individual gene trees (minimum evolution; all nucleotide sites) for three of the MLST loci used to produce figure 4. Sequences are coloured according to the species cluster in which they are present, as shown in figure 4.

Alongside *S. pneumoniae* and *S. pseudopneumoniae*, within the mitis group of streptococci, are the closely related named species, *Streptococcus mitis* and *Streptococcus oralis*. Isolates identified by API RapidID 32 strep as either *S. mitis* or *S. oralis*, when subjected to MLSA, fell into two clusters that were distinct from each other and from the other two species (figure 4). Each of these two clusters included strains identified as both species, presumably due to limitations in the API tests to identify them correctly. The names shown in figure 4 reflect the predominant species identification of the strains within the clusters, enabling us to define one as associated mainly with *S. mitis* strains and another as containing the majority of those identified as *S. oralis*. With the exception of one strain, all the ‘*S. oralis*’ strains were grouped together in 100% of trees drawn from the posterior probability at stationarity. Likewise, the ‘*S. mitis*’ group is found with 100% posterior probability, and is closely allied to the *S. pneumoniae* and *S. pseudopneumoniae* clusters. The topology of the four mitis group clusters differed, with substantially more diversity within the *S. mitis* and *S. oralis* clusters (average sequence diversity of 5.1 and 6.2%, respectively) than those of *S. pneumoniae* and *S. pseudopneumoniae* (average diversity of 1.1 and 3.0%). Furthermore, the average sequence divergence between the *S. mitis* and *S. pneumoniae* clusters was 5.8%, only slightly greater than that within the *S. mitis* cluster. It should be noted that this is not necessarily evident in the tree shown in figure 4 as a result of the best-fitting model of nucleotide substitution implemented across the tree. However, the diversity within the *S. mitis* and *S. oralis* clusters is further shown by the fact that each isolate examined had a different multilocus genotype. As in the *Neisseria* example, the individual gene trees completely fail to resolve the streptococcal species clusters identified using the concatenated sequences (figure 5).

## 7. Clusters: lineages or species?

In the two examples discussed in the previous section, MLSA resolves clusters that have a clear relationship to named species, even though recombination between the strains in different clusters is very apparent from the inspection of the trees obtained from the sequences of individual loci. Even in recombining populations that co-colonize the nasopharynx, clusters can be resolved using MLSA, which suggests that evolutionary forces have led to distinct non-overlapping genotypic clusters that the microbiologists have recognized as species. It remains to be seen whether greatly expanding the number of strains used in these analyses will maintain the resolution between the existing clusters and also whether including many examples of the less well-studied members of each genus will resolve clusters that support current species designations or will suggest new ones. Expanding the datasets can also change our current views of the clusters. For example, the identification of a group of strains that are much more similar to one of the *S. oralis* strains than to any of the others would produce a cluster of strains that are similar to each other and are clearly resolved from the other *S. oralis* strains. The phenotypic, biochemical and ecological properties of any such cluster can then be examined to see if a new species name is justified.

A major problem with the sequence-based approach to the definition of species is deciding whether resolved clusters should be considered to be different lineages within a species or deserve to be assigned species status. In the aforementioned examples, involving very well-studied groups of bacteria, clusters were correlated with the previous species designations; to be a useful taxonomic approach, MLSA needs to be capable of informing the division of large populations of poorly studied bacteria into species. Sequence clusters exist at all taxonomic levels, from the clusters of very similar genotypes that result from the diversification of a clone into a clonal complex (Feil *et al*. 2004) to deeper clusters that may be assigned as lineages within a species or as separate species.

With the MLSA approach, it may be difficult to decide the taxonomic level of a cluster and to distinguish those clusters that appear to be irreversibly set on different evolutionary trajectories, and which will continue to diverge from each other, from those that are best considered as different lineages of a single species. General criteria for recognizing the nature of clusters (e.g. the sharing or non-sharing of alleles, or the presence of fixed polymorphisms in different clusters) are probably not achievable for bacteria in which recombination may be very frequent or very rare. In the former case, allele sharing may still occur in clearly distinct species due to interspecies recombination, whereas if recombination is absent, allele sharing between distinct species or divergent lineages within a species is unlikely, raising questions of whether each clonal lineage should be considered genotypically to form its own species. These difficulties are not inherent weaknesses of the MLSA approach, but rather inherent problems in finding universal principles of species definition in the bacteria. The advantage of the MLSA approach is that it is pragmatic, rather than based on strict rules, and allows clusters to be identified and used as the basis for informed judgements on nomenclature, taking account of whatever additional data are available.

## 8. Species assignment on the Internet: electronic taxonomy

The MLSA approach appears to be a fruitful way forward, but to define the limits of clusters it requires the analysis of large populations of strains that cover the diversity within the genus (or part of the genus) of interest. The approach is ideal for collaborative groups with common interests, which can deposit their sequence data in a single database, along with strain characteristics, and can use their experience and knowledge of the genus to interpret the observed patterns of clustering of multilocus genotypes and to derive a consensus view of which clusters deserve species names. This process would take account of whatever additional phenotypic, biochemical, genomic, biogeographical or ecological information is available. Once the initial database has been established, and the clustering patterns have been used to guide the assignment of species, the concatenated sequence from any new strain can easily be compared via the Internet with a reference set that covers the diversity within each species cluster, to identify the cluster into which the strain falls and to assign its species or sub-species name. This facility is already available online at some of the MLST databases at www.mlst.net, for example to distinguish *S. pneumoniae* from *S. pseudopneumoniae* and *B. pseudomallei* from *B. thailandensis*.

New methods need to be accepted and used by taxonomists. The suggestion that MLSA may require the initial analysis of a thousand strains in order to uncover the patterns of clustering within a single genus, or even a part of a genus, and to assign species names, may appear daunting. The experience gained in the development of MLST for the characterization of isolates of bacterial pathogens provides encouragement, as three of the MLST databases now contain over 3000 isolates and several others contain well over a thousand. These databases have been built-up as collaborative ventures by academic microbiology, clinical microbiology and public health laboratories, many of which had no prior experience of sequence-based approaches, which share an interest in these important pathogens and which submit their data to the databases. The databases are large because MLST has proved its worth and has provided a gold standard for the precise and unambiguous characterization of strains of these pathogens. If the MLSA approach is similarly shown to be valuable, providing a much improved way of defining species within a genus, and of assigning new strains to species electronically via the Internet, there is little doubt that large curated databases can be developed for genera where there is sufficient interest. The great strength of the approach is that sequence-based taxonomy allows collaboration among laboratories with common interests and a pragmatic and consensual approach to defining species.

## 9. Concluding remarks

Sequence clusters are not of course necessarily species, but whatever other characteristic strains assigned to the same species should share, they should possess house-keeping genes that have similar sequences, and which are on an average more closely related to those from the same species than they are to those of other species (Ward 1998). We argue that clustering patterns should be the basis for defining species, by looking for natural discontinuities in the distribution of related genotypes, which may then be further elaborated upon, through classical phenotypic approaches. There may be situations in which large groups of related bacteria fail to form discrete genotypic clusters, and in such cases it is very unlikely that other taxonomic methods will give consistent species groups. If such situations occur it would seem likely that they would be within groups of bacteria, where there are continuing disagreements about their taxonomy.

The MLSA approach needs to be tested using large sets of strains that are considered to fall into a number of closely related species, so that the clustering patterns can be related to other information about the strains. This approach should be applied to sets of strains of related species that have high and low rates of recombination as different patterns of clustering are expected (Palys *et al*. 1997). The patterns of clustering need to be related to species designations obtained by the current polyphasic approach (Vandamme *et al*. 1996), and where they differ, the validity of the species divisions proposed by both the methods needs to be assessed, without assuming that new methods are only valid if they resolve the species divisions established with older methods.

The division of genera into species, and the development of simple phenotypic tests to recognize each species, has worked well in many cases; but in other cases, there have been constant taxonomic revisions and heated debate, which may reflect an underlying lack of clear species boundaries or methodological inadequacies. Single genes or single phenotypic tests are inadequate for reliable species identification in genera where recombination between species is relatively frequent and adds weight to the need for polyphasic approaches in taxonomy (Vandamme *et al*. 1996). The analysis of clustering patterns in well-studied genera should lead to guidelines in how best to apply the MLSA approach to define species within groups of similar bacteria that have not been well studied. In such cases, the identification of resolved clusters provides a basis for a search for biological correlates of the clusters, in terms of phenotypic, biochemical or ecological differences, or biogeography (Gevers *et al*. 2005).

A distinguishing phenotypic difference is required for the acceptance of a new species. If such differences are not found, groups of similar bacteria that appear to be genetically distinct have to be described by other terms (e.g. genomospecies). The requirement for a distinguishing phenotypic difference hinders the assignment of new species and needs to be reconsidered, as promiscuous recombination between closely related bacteria may prevent the identification of a single defining phenotypic difference, and the requirement for a phenotypic difference can be challenged if isolates can in future be identified online by using multiple house-keeping sequences to assign them to accepted species clusters by interrogation of a MLSA database.

One serious drawback, shared with other taxonomic approaches, is that MLSA cannot at present be applied to unculturable organisms, which are generally considered to be more numerous than those that can be cultivated (Rappe & Giovannoni 2003). However, defining the properties of culturable populations, and how they relate to differences in their 16S rRNA sequences, is only one way in which MLSA could help place this field on a more secure foundation.
